# Making Breath Visible: Reflections on Relations between Bodies, Breath and World in the Critical Medical Humanities

**DOI:** 10.1177/1357034X20902526

**Published:** 2020-04-27

**Authors:** Jane Macnaughton

**Affiliations:** Durham University

**Keywords:** breath, breathlessness, invisibility, lived body, medical humanities, narrative, sensation

## Abstract

Breath is invisible and yet ever present and vital for living beings. The concept of invisibility in relation to breath operates in concrete and metaphorical ways to extend ideas about breath and breathlessness across disciplines, in clinical spaces and in life experience. Using a critical medical humanities approach, I demonstrate that the poverty of narrative accounts and language for breath outside the health context have had a crucial influence enabling clinically mediated interpretations and accounts to dominate. These third-person accounts are important in the articulation of the ‘lived body’, but I balance this with a consideration of the subjective sensation of interoception, which has important implications for the visibility of breathlessness in both clinical and lay contexts. This article illustrates the rich potential of the subjects of breath and breathlessness within body studies and this special issue is a key step in making breath such an emergent topic.

## Why Is Breath Invisible?

Nothing is so much taken for granted as breath, the literal source of our lifeblood. Its importance and pervasiveness across the scales at which bodies interact with the materials, cultures and politics of life is perhaps a key reason for this. So entangled is breath in everything bodies are, experience and do that it gets taken for granted, lost from view and is rarely a direct focus of attention. Putting breath under the spotlight is, therefore, a daunting task. Attempting to unravel it from the mesh of significance which it has generated is challenging, but recognising its centrality at the outset is critical. What the cultural theorist, Steven Connor, has written of the air could so easily be said of breath:

How does one study an object that is everywhere? […] To study an object, one must pick it out from its surroundings, and concentrate it in one place. How was one to make of the air such an object? How was the air to be picked out of its surroundings, when air was ambience itself? How was the air to be brought before one when it was of necessity and at all times all about? (Connor, 2010)

‘Picking breath out from its surroundings’ is part of the task of this article, and I do this by arguing for the importance of breath as a focus for body studies. My task is made easier by focusing on situations where breath is a problem. Breath is not invisible to those for whom breathing is difficult, but their situation brings particular kinds of absence and isolation which are complex to address. The inability to breathe silences people, and polluted air shuts those with breathing difficulties more firmly behind the protection of closed doors, unseen and ignored by society. Invisibility is not, of course, the same as absence. Those whose breath is relatively absent feel invisible, and may even make themselves invisible because of a sense of shame at their condition. It is not that breath *is* invisible – it is going on within and around us all the time – but from many perspectives it may *appear* to be so.

I will open out some perspectives on breath through Scheper-Hughes and Lock’s three perspectives on the body, the ‘phenomenologically experienced body-self’, the social body focusing on ‘relationships among nature, society and culture’, and the ‘body politic, an artefact of social and political control’ (Scheper-Hughes and Lock, 1987). This approach offers a framework which structures an examination of the felt sense of being in a body that breathes or has difficulty breathing; how breath enables (or lack of it disables) interactions in the social sphere. I shall also examine other perspectives I consider somewhat underplayed in Scheper-Hughes and Lock’s account, mainly the ways particular cultures (clinical and literary) write about breath, the words they use and how this shapes experience of breathlessness. Having laid some groundwork to establish breath’s invisibility in a range of contexts and sites, I will go on to justify breath as a key emergent topic in the field of body studies. Breathing occupies an important space as a lens through which to understand the body, as a bodily function essential to the maintenance of life but one that, unlike the heartbeat, we can interrupt and control at will. The exploration of breath, therefore, speaks to key themes of importance to this journal: the distinction between objective biomedical measures and subjective individual perception of bodily sensations; and relationships between cognition, affect and interoceptive awareness (Paterson, 2019).

The research on which this article is based took place through the *Life of Breath*, a project characterised by interdisciplinary entanglement between humanities, social and clinical science researchers and ‘experts by experience’ (i.e. those living with breathlessness) articulating new questions and finding practical solutions to some of the problems identified. The disciplines involved in the project included philosophy, literary studies, history, anthropology, clinical medicine, neuroscience and a range of art forms, including visual art, dance and music. ‘Entanglement’ implies that the relationships between these disciplines are not just connections but in-depth conversations involving mutual understandings of the several ontologies and epistemologies involved. For example, pairing a philosopher trained in phenomenology with a phenomenologically minded anthropologist ensured that the fieldwork investigations of the experience of breathlessness went further than questions like ‘can you get up stairs?’ or ‘how does the weather affect your breathing?’. As I will demonstrate later in this article, engaging with clinical neuroscientists alerted us to new research on interoceptive awareness and the problems that might result from lack of such embodied awareness in people with breathlessness.

In common with previous contributions in this journal, the active term ‘entanglement’ also signals an approach to the body as a dynamic entity, ‘co-exist[ing] in shared ecologies’ (Blackman, 2010); co-existing and also co-evolving and co-responding. We have termed our approach ‘critical medical humanities’. Critical medical humanities champions transdisciplinary methodologies in attempting to understand health and illness, and desires to share findings and create new approaches that will enable knowledge of contextualised experience to influence the healthcare evidence base (Viney et al., 2015). What distinguishes the critical medical humanities approach is the integration of disciplines and methods, but also the emphasis on the groundedness of experience in local histories and the cultural record. This article, in common with the methods of critical medical humanities, draws upon a range of readings, analyses and some empirical work undertaken by members of the project team.

In attempting to bring breath into visibility I will treat it severally as sensation, means of interaction, political battleground and metaphor. Taking a critical medical humanities approach, I will demonstrate how the poverty of narrative accounts and language for breath outside healthcare contexts have allowed clinically mediated interpretations of the breathless body to dominate. I will explore this problem with reference to Latour’s concept of ‘articulation’ which acknowledges the importance of both objective and subjective perspectives in understanding the lived body (Latour, 2004), and how this is demonstrated through the notion of interoceptive awareness. I hope to illustrate how richly rewarding the topics of breath and breathlessness are for body studies, and their importance for developing further theoretical insights and practical solutions for those with breathlessness. This special issue is a key step in making breath such an emergent topic.

## Breath and the Lived Body

At the outset it is important to say what kind of a thing we are dealing with, and this is not easy to articulate. In a clinical sense, breathing, or respiration, is a physiological process involving the inspiration of oxygen and the expiration of carbon dioxide. Breath, therefore, consists of a collection of gases, variously constituted depending on the point in the respiratory cycle. For breathers, however, breath is more complex in its meanings than this, signalling as it does the presence and the passing of life; and the expression of emotion in gasps and sighs. It cannot really be captured in a moment, as a thing with a static existence, as it is constantly in flux, impermanent, continually changing its shape and character depending at what point in the process you happen to observe it. Breath can therefore be difficult to articulate or explain, but is central to the functioning of our bodies under both conscious and unconscious control.

This problem of articulation is one reason why knowledge of breath as a sensation is difficult to pin down. Breath is normally brought into awareness as a sensory phenomenon. Following Drew Leder’s approach we might think of the first-hand experience of breath as interoceptive (Leder, 1990: 39). Leder distinguishes three modalities of bodily sensation: interoception, which refers to sensing the internal activities of the body; exteroception, meaning the working of the five senses taking in the external world; and proprioception, providing information on the position of the body in space.

Leder’s key example of interoceptive awareness is of the gastrointestinal tract ingesting an apple. Applying his method to the conscious awareness of breath might go something like this. When we inhale consciously, paying attention to our breathing rather than leaving control to the autonomic system, we may be aware of the flow of air passing over our lips and tongue and flowing into our trachea. We cannot feel the flow of the air against the lung parenchyma, but we may be aware of the cold or heat of the packet of air we have just inhaled, and we can feel our chest stretching and expanding to a greater or lesser extent, then contracting as the whole process is reversed. Walking down a busy traffic-filled street there may also be the taste of polluted air in the mouth or a slight catch as the trachea attempts to filter dirt out from the air. We may also be aware of the expansion of the abdomen as the diaphragm contracts in inspiration and relaxes as we breathe out. In breathlessness, the process is rather taken out of our hands, with the autonomic system taking over to ensure the oxygen debt is repaid after running or exerting ourselves. In the case of a person with breathing problems, other parts of the physiological system are called into play through so-called accessory muscles, such as those of the neck. These may add an additional sensory experience to the exertion and work of these muscles supporting the chest wall in its bellows-like action.

Of course, most of the time breathing is in the background – it is invisible to the sensing body – but uniquely among internal viscera, the lungs are not part of what Leder terms the ‘recessive body’ (1990: 53), that is the body outside our conscious influence. We can take over control of our breathing, slowing it down, deepening it, even stopping it for a while. This control is essential for the activities of normal living, such as eating, speaking, coughing or sniffing, or specialist activities like singing, playing a musical instrument or freediving. The operations of conscious control add considerable complexity to what might determine the experience of breathlessness in normal and pathological states, rendering thoughts and feelings potentially much more influential.

First-person experience of breath and breathing is, then, deeply imbricated with aspects of the invisible: breathing being the function of hidden organs, sensed only in the movement of air through mouth and trachea, and only if part of conscious focus. The experience of breath and breathlessness are key sites of analysis for understanding the phenomenological notion of the ‘lived body’ (Leder, 1990: 7) because these sensations are under both conscious and unconscious control and slip in and out of awareness depending on context. Inspired by the writings of Merleau-Ponty, Leder sees the ‘lived body’ as an attempt to ‘escape from cognitive habits of dualism deeply entrenched in our culture’ (1990: 5). While our fleshly material first perceives the world, it recedes into the background as cognition and interpretation take over: ‘I am not in front of my body, I am in it, or rather I am it’ (Merleau-Ponty, 1962: 173). While proposing the foundational importance of the body as perceiver of the world, and the primacy of subjective experience, Leder also insists that the lived body involves a ‘third-person’ perspective, including the body ‘articulated by science as well as the life-world gaze’ (Leder, 1990: 7). Breathlessness is also subject to this ‘third-person’ perspective being observable and measurable by clinical technology, but also inscrutable since objective measures do not equate with lived experience.

This point is critical to what I will argue is the place of breath in body studies. Breath as sensory experience is difficult to explain; and in relation to the symptom of breathlessness, scientific explanations have become the dominant way of articulating the sensation. This has led to problems of so-called ‘symptom discordance’ where the felt experience of breathlessness does not equate with clinically measured lung function (Herigstad et al., 2011). I will return to this theme in the final section of the article in attempting to close the gap between these disaggregated first- and third-person perspectives incorporating some recent insights offered by research on interoceptive awareness into the dynamic interactions between body and world.

## Breath and the Social Body

As well as a first-person encounter with one’s own body, breathing is also a social phenomenon, and is experienced, and perhaps brought into greatest awareness, through interaction with others. These are often conscious activities dependent on breath, such as speaking, sighing, gasping and other expressions of emotion. Lande (2007) looks at how the socialisation of army recruits is consciously mediated by breath. According to Lande, breathing ‘properly’ is a necessary prerequisite for running in a pack, firing a rifle and even exerting authority: ‘it doesn’t look good as a leader if you are huffing it at the rear’ (2007: 100). Breathing is the activity that ‘coordinates bodies-in-time’; it is what signifies the ‘experience of the “We”’ (Lande, 2007). That universally shared experience of breathing, therefore, creates a common bond that we are often not aware of unless it is brought into visibility by activities that command attention, such as conversation or running in a pack (Lande, 2007).

Exclusion from that shared community of healthy breathers, when breathing becomes dysfunctional, can be an isolating experience. For those with breathing problems, social life is likewise difficult (Nicholls, 2003). Their breathing takes its own pattern and may be unpredictable and impossible to coordinate with that of others. Research on the *Life of Breath* project with British Lung Foundation (BLF) ‘Breathe Easy’ support groups has reported some of the kinds of arrangements group members make to avoid the embarrassment of being out of breath in the company of friends. Contrary to expectation, one group member reported that she does not accept lifts to social events, preferring to get herself to a venue in plenty of time to recover her breath before to greeting friends. It is common for people with breathlessness to avoid walking along the street accompanied as they find it difficult to walk and talk at the same time. Others reported using the street tactic of pretending to look in shop windows or use a mobile phone to recover their breath. The invisibility theme is particularly acute in this context because breathlessness (except in the most severely breathless) only becomes visible on exertion. One group member with pulmonary fibrosis remembered being told by an acquaintance: ‘you are looking really well’, and responding, ‘I am actually awaiting a lung transplant’. Another spoke of getting ugly looks from passers-by when parking his car in a disabled parking space, as it was only when he started to walk that his problem became apparent. As McGuire and Carel note, people living with breathlessness have a paradoxical attitude to its visibility. On the one hand, there is a desire to hide their problem for complex reasons associated with wanting to appear ‘normal’ in social contexts; on the other, there is resentment when people are unaware of it, because of its invisibility at rest (McGuire and Carel, 2019).

Breath is, therefore, an important mediator of social interactions. It is a shared, taken for granted, common denominator that connects people through the exchange of words and breath itself. In its absence, coordinated and in-common social life can become fractured because breath and words are not possible. The physiological and emotional rationale for this fracturing is only one element, however, and in the next section I will consider the interactions of politics and problematic breathing.

## The Breathless ‘Body Politic’

Breathless people are among the most marginalised in Western societies and one reason for this it that breathlessness disproportionally affects the most disadvantaged communities. A report by the BLF on the impact of lung disease in the United Kingdom (BLF, 2016) demonstrates the clear relationship between common lung diseases and socio-economic status. Chronic obstructive pulmonary disease (COPD) is currently the fourth and soon to become the third most common cause of death in developed countries (Mathers and Loncar, 2006), and those living in the most socially deprived areas are more than 2.5 times more likely than those in more affluent areas to develop the condition. Key reasons for this include smoking, which has a much higher prevalence in disadvantaged communities (Stringhini et al., 2010) and among certain groups marginalised by ethnicity and gender (Millward and Karlsen, 2011). Other influences also include outdoor air pollution. This much publicised global problem, highest in socio-economically deprived parts of cities like Delhi and London, contributes to the development or exacerbation of lung disease especially in the more vulnerable elderly and children (Marmot, 2010).

Partly as a consequence, it is uncommon to see someone out on the street struggling and having to stop to catch their breath, or walking along with an oxygen tank. The marginalisation that comes from socio-economic status is compounded by physical difficulty, but also by the fact that people with lung disease are often ashamed of their condition and do not *wish* to be seen. The word ‘invisible’ is a recurrent feature in articles describing the experience of breathless people. An article about people who suffer from COPD entitled ‘The invisibility of breathlessness’ shows how breathlessness is seen as ‘shameful’ and ‘embarrassing’, as self-inflicted because of smoking or though failing to preserve fitness in later life (Gysels and Higginson, 2008). The BLF’s 2007 report on COPD in the United Kingdom was likewise entitled ‘Invisible Lives’ (BLF, 2007). The report noted that individuals may feel invisible but there is also a problem of the so-called ‘missing millions’: the estimated almost 3 million people who suffer from COPD but do not seek a diagnosis because they do not recognise they have a lung problem. This lack of awareness is more profound in areas of social deprivation. People tend to accept the slow deterioration and chronic cough associated with conditions such as COPD as their lot, and do not present to health professionals. Even when they do see their GP and are offered support, the perception that they are ‘unworthy’ may contribute to lack of uptake of management options such as pulmonary rehabilitation (Harrison et al., 2014).

The often ill-informed perspectives of others on breathless people are important determinants of their invisibility in society. Dolezal, writing on the subject of shame, reflects on Sartre’s account of the ‘Look’, which ‘arises when one embodied subjectivity encounters another’ (Dolezal, 2015: 30). Dolezal’s point is that in encountering others, we see ourselves through their eyes as Sartre puts it (Sartre, 2003 [1958]: 329). The content of that ‘being seen’ is influenced by how society views aspects of ourselves that we feel may be stigmatised, such as body size, age, gender and also breathlessness.

The marginalisation of those who find breathing difficult is compounded by the invisibility of research funding. Research on cardiac disease and non-respiratory cancers receives around 10 times the funding for lung disease. This has consequences for mortality, which for heart disease has declined by 15% over the last decade in the United Kingdom, while lung disease has remained static (BLF, 2016).

What is apparent from my ‘three bodies’ account of breath and breathlessness is that the way society views people with breathlessness is dominated by a health-related narrative. We recognise that being out of breath is not entirely a function of illness, but can be associated with fitness and strength. However, the achievements of athletes are rarely celebrated in terms of breath, but rather by admiration of the visible appearance of their fit and supple bodies. Breath is invisible beside what our eyes can see. People with breathlessness for other reasons are invisible as individuals in our society and their story is told largely through the perspective of health. Multiple reports attest to their contribution to mortality figures, hospital admissions, winter bed crises (BLF, 2017) and costs to health services. It is unsurprising that the shame and lack of agency felt by people with lung disease is compounded by a public health narrative that is powerful by virtue of health’s inherent cultural and moral dominance, and an absence – not just invisibility – of other narratives. In the next section, I will establish the paucity of language in Western accounts of breathlessness, and even breath itself, and also discuss alternative narratives that have potential to contest this dominance.

## Breath in Language and Culture

Sartre emphasises the critical importance of language in understanding the body as seen by others:

Language by revealing to us abstractly the principle structures of our body-for-others (even though the existed body is ineffable) impels us to place our alleged mission wholly in the hands of the Other. We resign ourselves to seeing ourselves through the Other’s eyes; this means that we attempt to learn our being through the revelations of language. Thus there appears a whole system of verbal correspondence by which we cause our body to be designated for us as it is for the Other by utilizing these designations to denote our body as it is for us. (Sartre, 2003 [1958]: 377)

If this is the case, then the language of breath and breathlessness does not give us much support in ‘designating’ our body. As part of our research programme on *Life of Breath*, we hosted what we called ‘Breath Lab’: a research conversation involving a group of people with breathlessness, their relatives and carers, clinicians and policymakers discussing the language of breathlessness. By hosting this event outside any clinical context we hoped to capture some words and phrases that were not determined by diagnostic patterns and norms. The conclusion, however, was that that breathlessness was difficult to describe. One respondent commented on the fact that there are a range of words to convey the ‘character’ of pain, such as ‘sharp’, ‘dull’, throbbing’, ‘burning’. But when people are called upon to describe breathlessness it seems more difficult to objectify the sensation as something apart from the self which can be spoken about. Poetry written as part of a Writer in Residence programme we organised with a local ‘Breathe Easy’ support group revealed not only the problem of finding the words to express the experience of breathlessness, but also the lack of opportunity for such expression:

Grab, grasp with gratitude

this chance to speak.

To say what?

Can I do it?

Can we do it?

Do we have the courage?

Do we have the language?

We have the thoughts.

mostly hidden.

But words?

Denied, or rather not asked for

over the millenia.

From *A Chance* by Jill Gladstone (reproduced with permission from the author)

The reason for this void in the language of breathlessness is partly related to its existential inexpressibility (Carel, 2016) and to the fact that there are few accounts of either breath or breathlessness in non-clinical spaces. If we accord literary accounts in Western cultures the role both of recording and constructing our understandings of lived experience, the absence of breath from such a record is remarkable. Greek classical writers, widely regarded as the progenitors of many aspects of Western thought, wrote extensively on breath. Aristotle produced two treatises, *On Respiration* and *On Breaths*, and Plato’s cosmology as described in the *Timeas* has a long account of breathing. Yet we lack scholarly commentaries on breath in this context. Ideas about breath and its significance as the life force (*pneuma*) or vital spirit are also informed by religious texts, such as *The Bible*, in which God in the creation story creates man by breathing his breath into Adam. Such ideas provide resonances about the centrality of breath to life that are picked up in Shakespeare’s plays. In *A Winter’s Tale*, Leontes detects life in the stone statue of Hermione: ‘Still, methinks/There is an air comes from her. What fine chisel/Could ever yet cut breath?’ (Shakespeare, 1951). In the final scene of *King Lear*, the King calls for a mirror to detect breath on the lips of the dead Cordelia. Yet there are no academic writings on the subject of breath in Shakespeare.

Indeed, breath is a theme that few literary scholars have engaged with critically. One exception is Davina Quinlivan’s book, *The Place of Breath in Cinema* (2012). She is one of the few contemporary cultural theorists to recognise the importance of breath as a lens through which to examine culture and its societal impacts. Her focus on film as an audiovisual medium enables her to identify harsh or heavy breathing (such as that of Darth Vader in *Star Wars*; Quinlivan, 2012: 5) as a stigmatised cinematic trope reflecting danger and fear. Breath is most frequently to be found buried in other themes and dealt with tangentially through, for example, accounts of the air, such as Connor (2010) or Irigaray (1999); or miasmas or fogs, such as Corton (2015). As the authors of a new book produced by *Life of Breath* on breath in literature suggested: ‘Perhaps because breath functions so easily as an aesthetic substrate, it has been difficult to say anything substantial about it, in itself’ (Rose et al., 2018). It seems that breath is present in writing on voice, life, spirit, soul and body, but, with the notable exception of Quinlivan, it is never quite the focus of full attention, except when absent or difficult, as in the clinical context.

Anglophone literary culture, therefore, serves rather to compound shame and encourage invisibility for those who live with breathlessness. Literary accounts focus on the atmosphere of fear created by the sound of harsh breathing, on the sense of foreboding induced by the concealing effects of foggy air, or on breath as a metaphor for the suffocating effects of oppression. The themes of invisibility, concealment and shame overwhelm any accounts of breath as the giver of life or as an essential attribute for human existential connection.

## Breath and Articulating the Body

As a foundation for exploring the importance of breath in body studies, I have illustrated how breath is invisible for the individual body, the social body and the body politic, noting furthermore how breath’s absence from cultural accounts has led to an impoverished language of breath. This absence and impoverishment enables me to argue for breath as an important but neglected focus for body studies in how it emphasises the importance of balance and integration in ways of understanding the lived body.

Bruno Latour, in a previous issue of this journal, argues for the importance of ‘articulation’ as a way of having a body; of being alive:

An inarticulate subject is someone who whatever the other says or acts always feels, acts and says the same thing […]. In contrast, an articulate subject is someone who learns to be affected by others – *not by itself* [italics in original]. There is nothing especially interesting, deep, profound, worthwhile in a subject ‘by itself’, […] – a subject only becomes interesting, deep, profound, worthwhile when it resonates with others, is effected, moved, put into motion by new entities whose differences are registered in new and unexpected ways. (Latour, 2004: 219)

Latour dismisses the dualism of mind and body and instead conceptualises the body as ‘*an interface that becomes more and more describable as it learns to be affected by more and more elements*’ (Latour, 2004: 206, italics in original). What is on offer through this idea of ‘articulation’ of a body in the world is a way of integrating all the interactions that make a body a living entity, including the measurement instruments of the clinic, the cultural metaphors of the arts and – for breath – existential idea of the presence of a higher being or soul.

The problem that the example of breath makes clear is that clinical articulations dominate and define the lived experience of breathlessness. This has amplified the often discussed divergence between objective measurement and subjective experience of illness (Carel, 2018) which is often attributed to medicine’s reliance on technology. Such technology can skew the being of a breathless body towards the inarticulate subject Latour describes, leading to invisibility and silence. In this final section, therefore I wish to discuss how breath is articulated in the clinical context, and the consequences of this for people with breathlessness. I will go on to demonstrate that by widening this articulation to involve the dynamic relations between interoceptive awareness and experience it may be possible to reanimate the body through offering new means of articulation.

## Breath Articulated in the Clinic

The clinical context is marked by a drive to understand the mechanisms underlying sensation so that symptoms can be defined and labelled with a diagnosis (Johnson and Currow, 2015). The story of breathlessness has been one told largely in the language of physiology. Clinicians working in respiratory medicine have long been puzzled by the mismatch between measured lung function and experienced breathlessness (so-called ‘symptom discordance’) (Herigstad et al., 2011). Recent clinical updates on breathlessness acknowledge that the experience ‘derives from interaction among multiple physiological, psychological, social and environmental factors, and may induce secondary physiological and behavioral responses’ (American Thoracic Society, 2012). The clinical response to understanding the interactions between these factors is to turn to the brain. The new neuroscience of breath is starting to model this complexity thus bringing further insights into the discordance between mechanisms and the lived experience of symptoms.

Lansing et al. in a landmark paper in 2009 propose a multidimensional model taking its lead from pain studies (Lansing et al., 2009). Using functional magnetic resonance imaging (fMRI) they describe three distinct ‘separable qualities’ of uncomfortable breathing: ‘air hunger’, ‘the work of breathing’ and ‘tightness’. Having described these sensory qualities and linked them to distinct physiological mechanisms, the authors move to the affective qualities of breathlessness distinguishing between sensory intensity and affective intensity (A_1_) or unpleasantness. They further distinguish immediate experience of unpleasantness (A_1_) and a subsequent stage of cognitive evaluation and emotional response (A_2_). This later stage mirrors the model of chronic pain where (as with chronic breathlessness) negative emotions such as depression, anxiety and fear are common consequences.

There are, however, a number of problems with this model that obfuscate the lived experience of breathlessness. First, the authors connect people’s ‘incommunicable’ sensory experience to physiological mechanisms by offering word or phrase descriptors to their subjects and asking them to choose the best fit. As I have already noted, breathlessness is notoriously difficult to describe. This method is, therefore, very likely to be highly suggestible to patients and it may also lead to a narrow range of descriptors making it easier to ally them to discrete mechanisms or indeed to the researchers’ three ‘separable qualities’. In addition, as Hardie et al. (2000) have demonstrated, the language used by different ethnic groups may differ even when apparently describing the same sensation. When asked about this the authors acknowledged that their experimental subjects tended to be young, white college students (personal communication). What is also striking about Lansing et al.’s model is that the A_2_ emotional response is proposed as deriving *from* the sensation of breathlessness. But what seems clear is that the way people feel about their chronic breathlessness profoundly colours how the sensation is perceived (Hayen et al., 2013). The problem with this articulation is that clinical experiments are largely carried out on normal subjects whose bodies and minds have not been subjected to years of chronic breathlessness, and its consequent dynamic effects on physiology and neural mechanisms. Real patient studies are required, but those patient studies are challenging for people whose condition does not enable them to spend time lying flat in the enclosed tunnel of an MRI scanner.

This example illustrates the powerful influence clinical culture has not only in determining the language and metaphors of breathlessness, such as ‘air hunger’, but also how the drive to atomise sensory mechanisms through the use of apparently objective imaging technologies such as fMRI can further obscure the lived experience and sensation of breathlessness. Braun reveals the racial politics of the spirometer, another key machine for measuring breathlessness (Braun, 2014 ), and Dumit (2004) notes how positron emission tomography scans of the brain have become shorthand for different kinds of person: ‘normal, schizo, depressed’ (p. 6). Our Western cultural obsession with the image tends to fix bodies in time and space suspended in a web of sometimes suspect clinical assumptions.

If we move away from the static images that confine the lived body within the parameters of what clinical technology might objectively describe, but which are unable to take into account the dynamic interplay between body, mind and experience, we can start to understand the origins of so-called ‘symptom discordance’ in breathlessness. As Leder says, ‘in the West, the body (even the body interior) is largely thematized *qua* external objects, that thing which can be opened on the pathologist’s table or imaged through magnetic resonance imagery (MRI)’ (Leder, 2019: 314). Leder, like Latour, also acknowledges the importance of this ‘third-person’ perspective (Leder, 1990: 7): the body as ‘experiencer and experienced’ (Leder, 2019: 307). The subject of breath and the experience of breathing, however, enable a deeper exploration of the body as experiencer through a focus on interoceptive awareness, which has recently become a key field of interest for both scientists and philosophers (Tsakiris and de Preester, 2019).

In this final section, I will now return to the initial theme of this article – that of the sensing body. The example of breath indicates how that invisible body-self might be more effectively articulated through an examination of interoceptive awareness. This concept enables exploration of the complex relationship between subjective and objective body-self and also points to the dynamic potential for bodies (even sick bodies) to change and develop.

## Breath Articulated from the Inside: Interoceptive Awareness

Recognising the importance of affect and cognition, there is now a move in neuroscience to take the dynamic relationship between body, brain and world into account. This is clearly exemplified in the case of breathlessness, as Hayen et al. acknowledge:

Replicating the emotional component of dyspnea [breathlessness] in a laboratory environment is difficult as laboratory dyspnea does not cause the existential fears dyspnea sufferers encounter in daily life, hence patient studies will be necessary in order fully to comprehend all aspects of dyspnea. (Hayen et al., 2013)

In the clinical context, however, this ‘emotional component’ tends to become reduced to ‘anxiety and depression’ (American Thoracic Society, 2012: 437) because these are recognised aspects of chronic disease for which a range of validated clinical measuring instruments have been developed. The high prevalence of anxiety and depression in people with chronic breathlessness has also been associated with poor interoceptive awareness (Garfinkel et al., 2016).

For those with breathing problems, breathlessness is a constant and unwelcome accompaniment to life, and exercise – which makes it worse – is on the whole to be avoided. Oxley’s work on *Life of Breath* has revealed that many with breathlessness seek ‘safe spaces’ where they are not called upon to challenge themselves with movement. One respondent said:

Where am I most comfortable? In my chair. [laughing, coughing] – I’m not happy when I’m not in my chair. Then I’m just puffing and wheezing.

Under these circumstances, it is not surprising that those with chronic breathlessness have poor interoception. The body is a burden best ignored, and staying inactive, in order not to notice it, the optimal state of being. This tendency, along with the dampening effect of emotional states, is likely to contribute to the problem of symptom discordance in breathlessness (van den Bergh et al., 2019: 213).

This ‘problem’ of discordance arises because the fully articulated ‘lived body’ is not acknowledged in clinical contexts. What both Leder, in his characterisation of the ‘lived body’, and Latour, in his focus on ‘articulation’, assert is the critical importance of first- and third-person subjective and objective perceptions of the body in order fully to realise the body-self. It is here, I think, at the point of articulation of these distinct perspectives, that the sensory modality of interoception can help act as an explanatory mechanism for why these perceptions act in different ways, and also mediate between them.

Three dimensions of interoception have been identified as ‘distinct and dissociable’ in neuropsychological terms (Garfinkel et al., 2015). These dimensions are interoceptive accuracy, sensitivity and awareness. These mean, respectively, a measurable ability to detect (for example) your own heartbeats; the self-evaluation of your own ability to do so, and a ‘metacognitive’ ability to gauge and judge your own awareness. These different dimensions appear to drive a wedge between the two perspectives of the subjective lived body and the objectively assessed body in the context of the clinic. The notion that there is an ‘accurate’ or ‘inaccurate’ way to perceive your own bodily sensations seems not to make sense. However, this apparent lack of articulation enables us to rethink the idea of how bodily symptoms are assessed.

Van den Bergh and colleagues reflect upon the idea of accuracy in relation to symptoms (van den Bergh et al., 2019). They note that in the clinical context the idea of symptom assessment and consequent diagnosis depends upon the idea of accuracy. The assumption is that patients report symptoms that are the direct effect of some physiological dysfunction detectable through clinical tests or imaging. The ‘assumption’ here is that the patient’s interoceptive awareness is accurate and represents a true assessment of the underlying pathophysiology. Symptoms themselves are rarely the focus of clinical interest, only what they apparently have to say about the physical state of the body. Van den Bergh et al. suggest that this ‘accuracy assumption’ represents a ‘fundamental implicit contract among the patient, the physician, and the healthcare system’ (van den Bergh et al., 2019: 213). However, this only works under circumstances when the relationship between physiological stimulus and perceived symptom is simple. In the context of chronic, multisystem conditions, with complex sets of stimuli across a range of bodily systems (like breathlessness), the relationship tends to break down, giving rise to so-called ‘discordance’. However, when we relate symptom and clinical assessment in a different way, such as by assessing a person’s perceived (i.e. interoceptive sensitivity to) breathlessness in relation to risk of dying, we get a different story. It turns out that the *perception* of breathlessness is a better predictor of mortality than objectively measured breathlessness (Nishimura et al., 2002). This suggests that the coming together, or (in Latour’s sense) articulation, of physiological state with social life, past history, emotion and cognition is critical to prognosis in relation to complex symptoms like breathlessness. How you feel about it, how it has affected your life overall and what impact it has had are as important as how well your body is objectively functioning. As Noga Arikha has it: ‘interoception lies at the core of our very sense of self: physiology and mental life are dynamically coupled’ (Arikha, 2019: 3).

The critical term here is ‘dynamically’. This aligns with the phenomenological sense that that our minds and bodies are in continual interaction with the ways in which the individual experiences life. For the anthropologist, Mark Nichter, ‘embodiment is a dynamic process’ wherein past, present and future experiences are in active interplay with how bodily sensations are perceived at any one time (Nichter, 2008). Tim Ingold and Gisli Palsson speak not of human beings but of human ‘becomings – that is not as discrete pre-formed entities but as trajectories of movement and growth’ (Ingold and Palsson, 2013: 8). Entangling these ideas with recent theories about how the brain works through the Predictive Coding hypothesis, it is also clear that neuroscience proposes a dynamic relation between mind, body and time (Knill and Pouget, 2004). Predictive coding suggests that in order to deal with the complexity of living in a world of sensory uncertainty, we make predictions about sensory information on the basis of previous experience (unconscious ‘priors’). These predictions are unconsciously generated but enable us to infer how we will perceive sensory stimuli without having to compute the full range of information coming into our brains. Unconscious prior experience may be embedded in our brains along with conscious habits of thought, and responses may be established over years of living with a symptom like chronic breathlessness, but the very fact that they are established in this dynamic way, through interaction between body and world, means they are not set in stone.

What a study of breath brings here then is an ability to articulate objective scientific and subjective views of the lived body through the medium of interoceptive awareness. As I said at the outset, breath is under both conscious and unconscious control: we can control our breathing though our brain may override this control if the body is at risk. Breath is therefore partly of the ‘recessive’ body, traditionally the preserve of bioscience to describe, but at the same time of the body as experienced and described subjectively. This means that breath is a unique sensation and function that can demonstrate how apparently disparate fields of understanding of the lived body come together. Those fields include those understood through subjective understanding, through the media of qualitative examination, and through cultural expression; and also the lived body understood objectively through clinical, neurophysiological analysis, and through neuroscientific theory. From these very different standpoints both agree that the lived body is a dynamic entity, shaped by the past and open to new articulation through future experience.

Through this articulation it is possible to see how symptom discordance arises if there is, as in the clinic, a focus on objective means of measuring breath. The dynamic potential of the lived body signalled through both objective and subjective views suggests that there is the possibility for change and positive adaptation to the subjective condition of breathlessness through the medium of interoceptive awareness. This sense of potential can provide hope for those suffering from breathlessness who may feel neglected and lost. Leder talks of gaining, or recovering, ‘inside insight’, arguing that greater awareness of the internal sensations of the body might divert us from actions that endanger health (Leder, 2019: 318). One approach that has found some efficacy in achieving this reconnection is dance (Philip et al., 2019). Dance movement has the potential to reconnect the breathless person with their body in ways that may allow the body to be experienced as expressive, and as aesthetically valued, rather than as a burden to be ignored. Interoceptive awareness – which is continually shaped and interpreted, consciously and subconsciously, by life experience including culture, imagination, social relations and religious belief – might help regain a sense of control over the uncontrollable and terrifying experience of breathlessness.

## Conclusion: Laying Breath Bare

This article has described the range of ways and contexts in which breath is hidden to individual bodies, for social bodies and the body politic. It has done so by looking at how breath is expressed in language and represented in clinical cultures and technologies. I have illustrated the potential harm this poses to those whose lives are afflicted by breathlessness, through the cultures of public health and clinical science, both of which tend further to obscure the lived experience of breathlessness. I have illustrated the absence of alternative accounts of breath and suggested that healthcare-related narratives have occupied this void providing society with the words and accepted explanations for how breath is understood and experienced.

Breath, as both a component of the ‘recessive’ body and the body under conscious control, is a unique lens through which to examine the intimate and dynamic interrelation of body and experience. Breath is perceived through interoceptive awareness, and that perception is in turn profoundly influenced by concurrent emotional and pathological states, such as anxiety and depression, common accompaniments to chronic breathlessness. Breathlessness is a key bodily symptom that demonstrates how clinical measurement can often be at odds with experience. A focus on interoceptive awareness enables us to make sense of this discordance as it signals the dynamic interrelations of the body and world which constitute the lived body, and which bring together subjective and objective views of the body in neuroscience and anthropology. Moreover, from a clinical perspective, these insights demonstrate the potential for change and development in ways that might actually help people with the lived experience of breathlessness come out into the open and find new ways of articulating with the world through language and movement. Critical medical humanities projects like *Life of Breath*, that not only seek to explore the experience of breathlessness in contexts outwith the clinical but also to entangle these literary, phenomenological and ethnographic explorations with clinical science, hold promise.
